# Rheological Properties Related to Extrusion of Polyolefins

**DOI:** 10.3390/polym13040489

**Published:** 2021-02-04

**Authors:** Evan Mitsoulis, Savvas G. Hatzikiriakos

**Affiliations:** 1 School of Mining Engineering and Metallurgy, National Technical University of Athens, Zografou, 15780 Athens, Greece; mitsouli@metal.ntua.gr; 2 Department of Chemical and Biological Engineering, The University of British Columbia, Vancouver, V6T 1Z4 BC, Canada

**Keywords:** shear viscosity, cross model, K-BKZ model, temperature-dependence of viscosity, pressure-dependence of viscosity, slip at the wall

## Abstract

Rheological properties related to the extrusion of polyolefins are the shear viscosity, the elongational viscosity, the slip velocity and their temperature- and pressure-dependencies. These properties are measured in the rheology lab mainly via a parallel-plate rheometer and a capillary rheometer. Then appropriate rheological models have to be used to account for all these properties. Such models are either viscous (e.g., the Cross model) or viscoelastic (e.g., the K-BKZ model). The latter gives the best fitting of the experimental data and offers excellent results in numerical simulations, especially in extrusion flows. Wall slip effects are also found and measured by rheometric flows. Modeling of extrusion flows should make use of appropriate slip models that take into effect the various slip parameters, including the effects of shear stress, molecular characteristics, temperature and pressure on the slip velocity. In this paper the importance of these properties in extrusion are discussed.

## 1. Introduction

Extrusion lies in the heart of polymer processing. Essentially nearly all polymer processing operations need an extruder to melt, mix and process polymers and their compounds [1,2]. To understand and optimize an extrusion process, the rheological properties first have to be understood [3]. In other words, it is difficult to understand and optimize a polymer processing operation without having first a thorough understanding of the rheological behaviour of the material under process over a wide range of time and length scales. Moreover, using the rheological properties in both shear and extensional flows, an appropriate constitutive equation should be identified, capable of capturing the correct rheological response of the material in both shear and extensional flows, including simple contraction flows through capillaries and slit dies [1,2,3,4].

In this article some important rheological properties related to extrusion are discussed. These include the following: (i) the entry pressure in capillary extrusion important to capture the extensional behavior of polymer melts [5,6,7,8,9,10]; (ii) the effects of temperature and pressure on the rheological properties [11,12]; and (iii) the slip behaviour of polymers at solid boundaries [13,14,15,16,17,18]. The main focus will be polyolefins as there is a considerable body of rheological data allowing the assessment of the importance of rheological properties in extrusion.

This paper is organised as follows. First the temperature sensitivity of the viscosity of polymers is discussed by means of experimental data obtained from a rheometer equipped with a parallel-plate geometry (Section 2). Next capillary flow is discussed (Section 3) as an important rheological test (i) to capture the effect of pressure on viscosity (Section 3.1) and (ii) to determine the entry pressure drop from the reservoir to the capillary die and use of this to evaluate the suitability of a rheological constitutive equation to predict it (Section 3.2) as well as to capture the important effects of extensional viscosity (Section 3.3). Finally, the possibilities of polymer melt wall slip are discussed and a comprehensive slip velocity equation is presented (Section 4) as an appropriate boundary condition for high-shear flows. 

## 2. Parallel-Plate Rheometry: Effect of Temperature

Parallel-plate rheometry is the starting point in any rheological study of polymer melts which can provide useful information on the structure–property relationships of polymers, i.e., effects of shear rate, molecular weight and its distribution, the complex and absolute viscosity of polymers in both shear and extensional flows [1,2,3,4]. Performing rheological tests at various temperatures and using the powerful technique of time-Temperature Superposition (tTS), the rheological properties can be captured over a wide range of time and length scales [1,2,3,4]. Consequently, using these rheological properties, the effects of temperature and pressure can be assessed easier, i.e., comparing them with rheological data obtained from pressure-driven flow such as capillary flow (discussed extensively below).

An important parameter that can be obtained accurately from the parallel-plate geometry is the effect of temperature on the rheological properties, which can be significant. The effect can be obtained by measuring the viscosity *η* of the melts over a wide range of temperatures *T* and then using the following equation to model the temperature shift factor, *a_T_*, determined from the application of the tTS to obtain the master curve of the viscosity at the reference temperature, *T_ref_*:

(1) $$a_{T}(T)=\frac{\eta }{\eta_{0}}=\exp\left[\frac{E}{R_{g}}\left(\frac{1}{T}-\frac{1}{T_{ref}}\right)\right].$$

In the above, *η*_0_ is the viscosity at the reference temperature *T_ref_*, *E* is the flow activation energy constant, *R_g_* is the ideal gas constant, and *T_ref_* is a reference temperature (in K). Typical values reported for the activation energy of various polymers can range from as low as ≈22,800 J/mol for HDPE to ≈83,000 J/mol for HDPE to for LDPE (depending on the degree of branching) to ≈116,500 J/mol of polystyrene [19]. This roughly corresponds to a decrease of viscosity of 36% to 370% by increasing the temperature by 20 °C, a nontrivial effect.

## 3. Capillary Rheometry

Capillary rheometry is extensively used in both industry and academia to assess the rheological behaviour of polymer melts at high shear rates as well before testing their processability in full industrial scale [1,3]. When such a flow is used and the raw data are collected, a number of important corrections should be applied before the rheological data can be compared with corresponding data from a rotational rheometer [1,3]. Details of analysis of raw experimental data obtained from capillary rheometer to calculate fundamental rheological quantities such as shear stress and shear rate can be found in most books on rheology [1,3]. In this paper, the importance of entry pressure is discussed. However, the important effects of temperature (discussed above) and pressure on viscosity should be considered next before the importance of entry pressure is discussed.

### 3.1. Effect of Pressure on Viscosity

The effect of pressure on the viscosity is very important for polymer melts. In extrusion operations, typically large pressures are encountered at high shear rates which can cause significant increase of viscosity due to pressure. This also causes significant viscous dissipation, which should also be taken into account, i.e., considering the energy equation in modeling essential non-isothermal flows [1,3]. 

The effect of pressure on viscosity can be studied by using capillary data from dies of various length-to-diameter (*L/D*) ratios [1,3,20,21,22,23,24,25]. As a first approximation, the following expression (Barus equation) can be used to determine the parameter, $\beta_{p}$, known as the pressure coefficient of viscosity:

(2) $$\eta_{p}=\eta_{0}\exp\left(\beta_{p}p\right)$$

where $\eta_{p}$ is the viscosity at pressure *p*, and $\eta_{0}$ is the viscosity at ambient pressure and reference temperature *T_ref_*. Combining Equations 1 and 2, the viscosity (or any other rheological property) can be modeled by

(3) $$\eta_{p,T}=\eta_{0}\exp\left(\beta_{p}p\right)\exp\left[\frac{E}{R_{g}}\left(\frac{1}{T}-\frac{1}{T_{ref}}\right)\right]$$

where now $\eta_{p,T}$ is the viscosity at pressure, *p*, and temperature, *T*, with respect to the viscosity of the melt $\eta_{0}$ at the reference temperature *T_ref_* and ambient pressure.

Using a pressurized sliding-plate rheometer, Koran and Dealy [26], Park and Dealy [27], and Park et al. [23], have determined the coefficient $\beta_{p}$ for various systems, including low-density polyethylene (LDPE), poly-*a*-methylstyrene-co-acrylonitrile (P*a*MSAN), and linear low-density polyethylene (LLDPE). For the LDPE (of main interest in the present work) a value between $1.3\times 10^{-8}$ Pa and $4.9\times 10^{-8}$ Pa has been reported by various authors [21,25,28,29]. This coefficient has also been reported to be a function of the shear rate, with $\beta_{p}$ of LDPE decreasing significantly with shear rate [3,30]. These typical values can increase the viscosity significantly. For example, for a typical pressure of 100 MPa in extrusion and a coefficient of the order of $10^{-8}\;to\;5\times 10^{-8}$ Pa, the viscosity can increase by a factor of $\exp(\beta_{p}p)$, that is 2.71 to 148 times, effects nontrivial.

### 3.2. Entrance Pressure Significance

**Figure 1:**
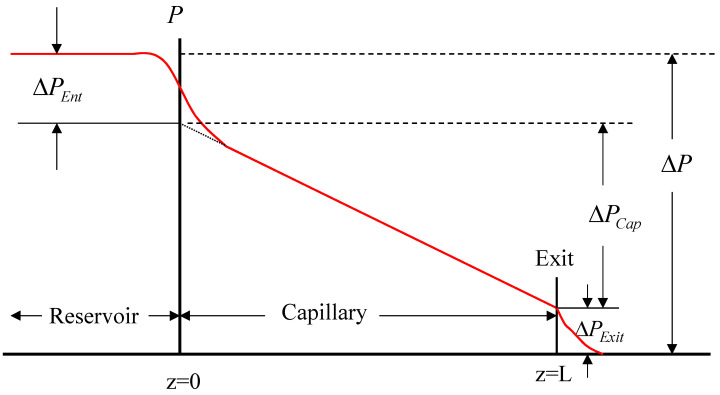
Schematic diagram of the entry and exit pressure losses in flow through a capillary rheometer (reservoir to die).

First, capillary flow involves flow through a contraction of a certain angle, where there is a large pressure drop associated with such flow, known as end (or entry or entrance) pressure [1,4]. Figure 1 plots the axial pressure variation in a capillary die including both its entrance and exit regions. It can be seen that the total pressure drop, Δ*p*, consists of three components and may be written as:

(4) $$\Delta p=\Delta p_{Cap}+\Delta p_{Ent}+\Delta p_{Exit}$$

where Δ*p* is the total pressure drop from the reservoir to the capillary exit, Δ*p_Cap_* is the pressure drop over the length of the capillary where the flow is fully developed, Δ*P_Ent_* the entry pressure which is mainly due to the extensional (acceleration) flow at the entrance, and the Δ*p_Exit_* the exit pressure associated with normal stress effects at the exit region of the capillary [3]. The combined end pressure $\Delta p_{End}=\Delta p_{Ent}+\Delta p_{Exit}$ is required in order to calculate the true shear stress. On the other hand, the entry pressure $\Delta p_{Ent}$ is frequently used to determine the apparent extensional rheology of molten polymers, a method well practiced in industry [5,6,7,8,9,10]. An appropriate rheological constitutive equation should be capable of capturing/predicting the entry pressure correctly, essentially capable of capturing the pressure associated with a simple contraction flow before tested in polymer processing where the geometries are much more complex (combinations of contraction and expansion of various degrees).

**Figure 2:**
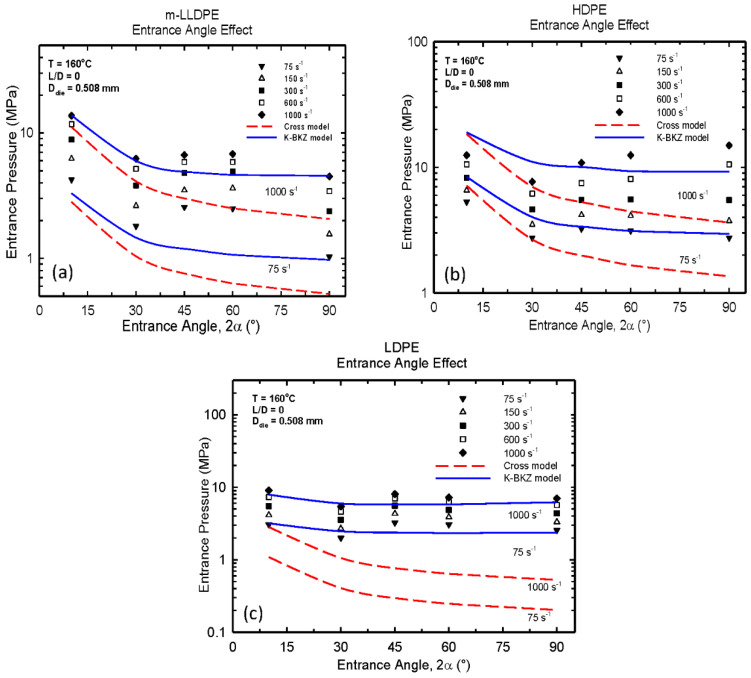
The entrance pressure of (**a**) LLDPE (**b**) HDPE and (**c**) LDPE at 160 °C as a function of contraction angle at various values of apparent shear rate.

Many studies have previously attempted to examine the origin of entry pressure and its prediction for various polyethylenes [31,32,33,34,35,36,37,38]. Significant under-estimation was reported in all these studies that have raised questions with respect to the appropriateness of the constitutive equations used and/or the adequate rheological data used to fit the model parameters, particularly those that control the extensional behavior of these polymers. The problem of predicting the end pressure for LDPE was solved satisfactorily at very high shear rates (up to 1000 s^−1^) for the first time by taking in to account the effect of pressure (mainly) and temperature on viscosity [39]. The authors pointed out the importance of considering in detail the significant effects of pressure and temperature on viscosity (including possible viscous dissipation effects) as well as the importance of extensional viscosity data to predict correctly the entry pressure. Typical results from the simulations are shown in Figure 2a–c for three polymer melts, a metallocene polyethylene (m-LLDPE), a high-density polyethylene (HDPE), and a low-density polyethylene (LDPE), respectively. Numerical results for the lowest and highest apparent shear rates of 75 s^−1^ and 1000 s^−1^ are only presented here for the sake of clarity. Several observations can be made: (i) the use of a viscous model (Cross model) significantly underpredicts the entry pressure as such models neglect the important viscoelastic and extensional rheological effects. On the other hand, the use of the K-BKZ model is capturing adequately well the entrance pressure as a function of the contraction angle for all three polymers. As the contraction angle increases, the extensional components are having a stronger effect, thus increasing the discrepancies between predictions from a purely viscous model and the measured experimental data [39]. Finally, comparing the entrance pressure between the three types of polyethylene, the authors concluded that it scales with the extensional viscosity of the polymer. For example, the LDPE (branched polyethylene) possesses the highest entrance pressure due to the significant strain-hardening effects originated from its branched structure.

### 3.3. The Importance of Extensional Viscosity

**Figure 3:**
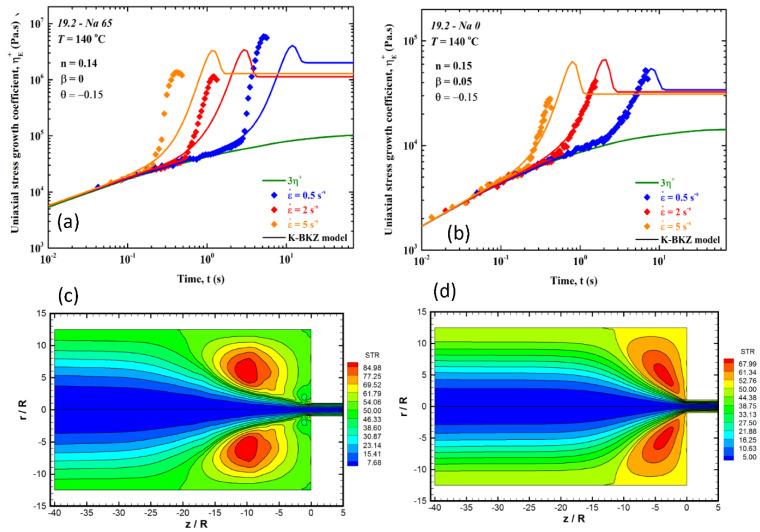
(**a**) The uniaxial tensile stress growth coefficient in start-up of uniaxial extension for sample 19.2-Na 65 at 140 °C; (**b**) the uniaxial tensile stress growth coefficient in start-up of uniaxial extension for sample 19.2-Na 0 at 140 °C. The continuous lines are fits of the K-BKZ/Wagner model to the experimental data. (**c**) Flow patterns (streamlines) for the copolymer 19.2-Na 65 at 140 °C for a die with *L/D* = 33 at the apparent shear rate of ${\dot{\gamma }}_{A}$ = 400 s^−1^. (**d**) Flow patterns (streamlines) for the copolymer 19.2-Na 0 at 140 °C for a die with *L/D* = 33 at the apparent shear rate of ${\dot{\gamma }}_{A}$ = 400 s^−1^. These figures clearly show that a higher extensional viscosity produces a larger and stronger vortex.

The importance of the extensional viscosity on the entry pressure was studied extensively for the case of ionomers [40,41,42,43]. Results are presented in Figure 3 for two polymers, one ionomer (19.2-Na65) and its corresponding copolymer (19.2-Na65). First, ionomers exhibit stronger strain-hardening effects, as can be seen from Figure 3a,b, respectively. The authors reported that the vortex size and intensity are increasing substantially with extensional viscosity, as can be seen from Figure 3c,d. These differences signify the strong effects of ionic interactions that give rise to strong strain-hardening effects. In general, the flow patterns (vortex size and intensity) scale with the number of ionic interaction (strain-hardening effects) in the case of ionomers. Similar conclusions can be drawn when a comparison between a linear and branched polymer is made [39].

## 4. Slip Effects

Unlike Newtonian fluids, polymer melts slip over solid surfaces when the wall shear stress exceeds a critical value [13,14,15,16,17,18]. In particular, slip effects have been reported in the capillary flow of molten polyethylenes [44,45,46,47,48,49], polydimethylsiloxanes [50], polystyrenes [51,52,53], polybutadienes [54,55], polypropylenes [56,57,58], fluoropolymers [59], polylactides [60], polyisobutylenes [61], ionomers [40,41] and other viscoelastic fluids [62,63]. Thus, in a comprehensive study of any melt, possible slip effects should be studied to be used as boundary conditions in high shear rate flows. This can be done by using at least three capillary dies having different diameters and the same *L/D* in order to keep the effect of pressure constant. If the flow curve shows a diameter dependency, the Mooney method can be used to determine the slip velocity as a function of the wall shear stress.

Many researchers have attempted to quantify the slip velocity of polymer melts as a function of wall shear stress, wall normal stress, temperature and pressure [13,14,15,54,64,65,66]. It has also been reported that the slip velocity increases with decrease of molecular weight for monodisperse polymers [52,53,54,63]. Moreover, it depends strongly on the breadth of the molecular weight distribution (MWD) with polydisperse polymers to slip more compared to their monodisperse counterparts at a given average molecular weight [63]. These effects should be taken into account as the relationships between slip velocity and molecular characteristics are nonlinear, and small changes in these parameters may result in large differences in the slip velocity.

A slip velocity model has been formulated based on reptation theory (presented below) to capture the molecular weight and MWD effects [66]. To this end, the formulated integral slip velocity model [66] was coupled with a fractionation model developed by van der Gucht et al. [67] to accurately capture the MWD effects, which in fact are strong for very broad molecular weight distribution [68]. Entropy driven migration of polymer molecules are known to occur, driving shorter polymers closer to the surface (surface migration/segregation), phenomena which have been predicted theoretically [69,70,71] and observed experimentally in polymer extrusion studies [72,73,74,75,76,77,78,79]. A comprehensive slip model that takes into effect these phenomena of segregation has been developed for polyethylenes [80] to predict the slip velocity of a large number of polyethylenes reported in [79,81]. 

As discussed above, using elements from the theory of double reptation, Ebrahimi et al. [68] and Najm and Hatzikiriakos [80] developed a slip velocity model that relates the wall slip of polymers, *V_S_*, with their detailed molecular weight distribution (MWD). This can be written as:

(5) $$a_{T}a_{p}V_{S}=A\left\{\int_{0}^{\infty }\left[M^{\beta }w(M)\int_{M}^{\infty }w(M^{\prime })dM^{\prime }\right]dM\right\}\sigma_{w}^{1/n}=Af(M)\sigma_{w}^{1/n}$$

where *n* is equal to the local slope of the flow curve of the corresponding polymer,$n\equiv d(\log(\sigma_{w}))/d(\log({\dot{\gamma }}_{w}))$, $\sigma_{w}$ is the wall shear stress, ${\dot{\gamma }}_{w}$ is the wall shear rate, *M* represents the molecular weight, $a_{T}$ and $a_{p}$ are the temperature and pressure dependency coefficients of slip respectively, and *A* and *β* are constants which depend solely on the polymer type and are equal to 1.0 × 10^10^ and −2, respectively, for HDPEs. This model gives the slip velocity of polymers based on the molecular weight distribution of the polymer in the bulk. However, fractionation phenomena are occurring during flow and such effects are important to be considered. Thus, given the molecular weight of the polymer in the bulk, *w_b_*(*M*), a model is used to calculate the molecular weight of the polymer at the wall surface, *w_s_* (*M*), that controls the slip of the polymers. Essentially the molecular weight at the surface, *w_s_(M)*, is related to that at the bulk, *w_b_*(*M*), by:

(6) $$\frac{\nu_{ex}\left(M\right)}{w_{b}\left(M\right)}=A_{C}\left(1-\frac{M}{M_{w}}\right)\pm A_{F}\sqrt{\left|1-\frac{M}{M_{w}}\right|}{\left(\frac{\sigma_{w}}{G_{N}^{o}}\right)}^{\frac{1}{n}}$$

(7) $$w_{s}\left(M\right)=w_{b}\left(M\right)+\nu_{ex}\left(M\right)$$

where $\nu_{ex}(M)$, $w_{s}(M)$ and $w_{b}(M)$ represent the excess, surface and bulk weight fractions and $G_{N}^{o}$ is the plateau modulus. The first term in Equation (6) represents the entropy driven migration while the second represents the flow induced migration effects. More details can be found in [46,80].

**Figure 4:**
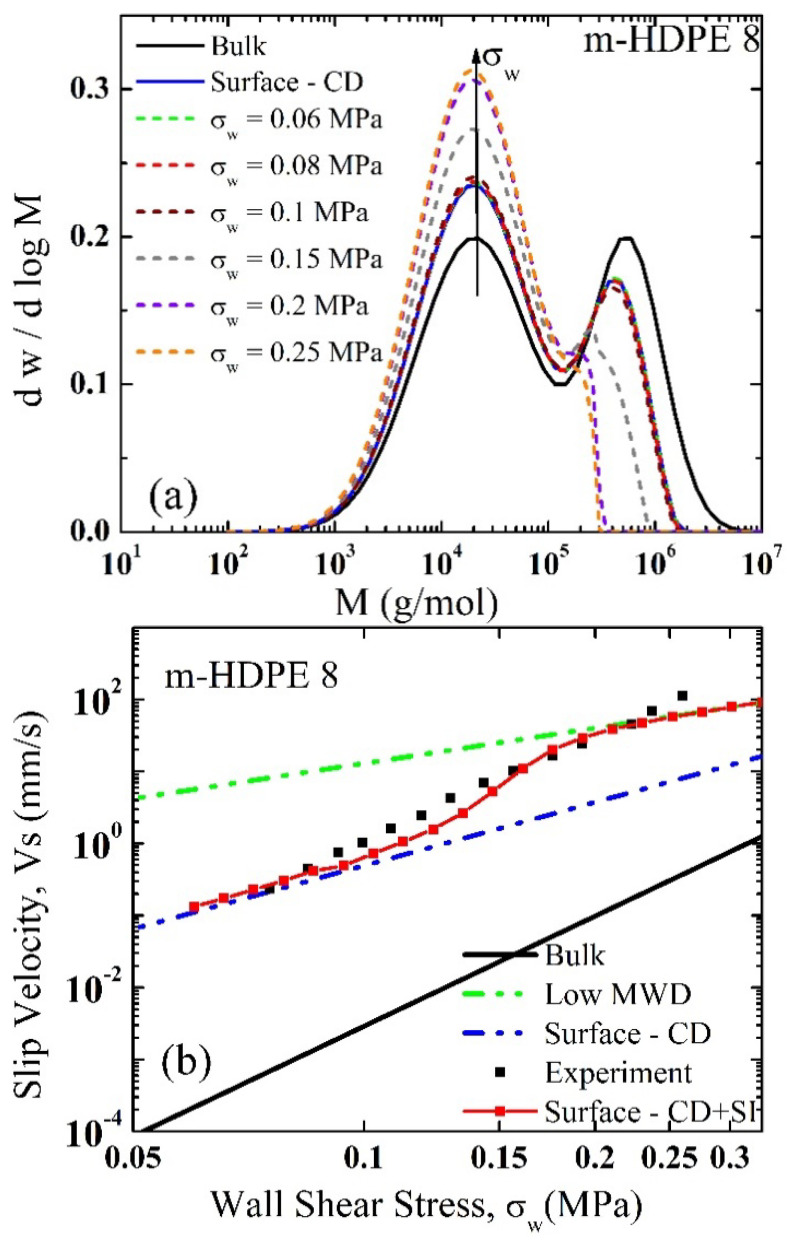
(**a**) The MWDs of bulk (solid line), surface-CD (concentration driven migration given by Equation (6) with only the first term), and the combined surface and shear-driven migration at various wall shear stress values (Equation (6)); (**b**) the slip velocity of m-HDPE-8; the experimental results (black squares), the bulk and low MWD are calculated based on Equation (5) with no migration effects, the concentration driven migration (surface CD) (Equation (6) only the first term) and the combined concentration and shear-driven migration (CD+SI) slip velocity (red squares connected with a continuous line) using Equation (5) coupled with Equation (6).

Figure 4 presents typical results comparing experimental slip velocity data for a PE melt with model predictions considering various cases. Figure 4a presents the MWD of the polymer and the various MWD of the polymer at the surface under the influence of various levels of the wall shear stress. As the wall shear stress increases essentially the population of smaller molecules increases at the surface at the expense of larger ones. Using the MWD of the polymer at the bulk, the prediction of the slip velocity (Equation (5)) is several orders of magnitude less than the experimental data as can be seen from Figure 4b (solid black line). Considering now the shear-induced segregation effects, which are represented by the first term only of Equation (6), the model prediction is significantly improved, essentially capturing the experimental data at small values of the wall shear stress. However, it underpredicts the experimental data at higher wall shear stress values, which indicates that the additional effects of wall shear stress (shear-induced migration) need to be included. When the concentration gradient effects as given by Equation (6) are taken in to account (entropy driven and flow-induced) the calculations (red squares connected with a continuous line) agree remarkably well with the experimental data (black squares). Note that the surface MWD depends on the value of the wall shear stress (several distributions are plotted in Figure 4a). As the wall shear stress increases, high-MW species are depleted from the surface at the expense of short-MW ones. As a result, the slip velocity increases further in a nonlinear fashion. 

## 5. Conclusions

Rheological data were considered from parallel-plate and capillary rheometers and discussed in the context of their importance in extrusion flows [1,2,3,4]. These include steady shear viscosity and transient elongational viscosity data and their temperature and pressure dependency, which should be used to best fit the parameters of an integral constitutive equation of the K-BKZ type. This integral model successfully simulates remarkably well the entrance pressure drops polymers are showing, and it is a suitable equation to be used in more complex flows such as extrusion. It is further capable of simulating successfully the flow patterns in the contraction areas in capillary dies showing that it captures the important effects of the extensional viscosity.

However, to successfully simulate complex flows of polymers in extrusion, the wall slip should be taken into account which seems to be the rule and not the exception. A comprehensive slip velocity model has been presented that is capable of capturing not only the dependence of slip velocity on wall shear stress and molecular characteristics of the polymers but also the possible strong segregation effects that occur at solid boundaries due to (i) entropy driven migration of smaller molecules towards the wall at the expense of larger ones and (ii) the flow induced migration effects that contribute significantly as well. Coupling of the slip velocity model with the segregation/fractionation model provides an appropriate boundary condition suitable to simulate viscoelastic fluids in complex flows.

This article pointed out the importance of some rheological parameters that are needed to improve the capabilities of rheological constitutive equations to simulate complex flows related to polymer processing operations such as extrusion. The K-BKZ model has been shown to be one of the most successful constitutive equations to simulate complex flows. It needs a minimum number of rheological tests to determine its parameters. Therefore, more work in this direction is recommended in the future, i.e., use of K-BKZ to simulate extrusion operations of polymers, polymer blends and polymer composites [82,83].
